# Ultrasound-guided modified thoracoabdominal nerve block for postoperative analgesia in laparoscopic renal cyst decompression: a randomized double-blind controlled trial

**DOI:** 10.3389/fmed.2025.1582428

**Published:** 2025-07-04

**Authors:** Mengning Wan, Jun Dong, Ke Wei, Juying Jin, Jun Cao, Baohong Yuan

**Affiliations:** The Department of Anesthesiology, The First Affiliated Hospital of Chongqing Medical University, Chongqing, China

**Keywords:** modified thoracoabdominal nerve block, laparoscopic renal cyst decompression, postoperative analgesia, ultrasound, randomized controlled trial

## Abstract

**Background:**

Laparoscopic renal cyst decompression (LRCD) is a common procedure in urology, but postoperative pain remains a significant challenge. While regional nerve blocks provide more targeted pain relief, there is no universally accepted pain management strategy for LRCD. The ultrasound-guided modified thoracoabdominal nerve block (M-TAPA) may offer effective analgesia by blocking the anterior and lateral branches of the intercostal nerves (T5-T12). However, its efficacy in LRCD has not been thoroughly evaluated.

**Objective:**

This study aimed to assess the efficacy and safety of unilateral M-TAPA in reducing postoperative pain and opioid consumption in patients undergoing LRCD, and to evaluate its potential benefits in enhancing recovery.

**Methods:**

In this randomized, double-blind, controlled trial, 61 patients undergoing LRCD were assigned to either the M-TAPA group (*n* = 31) or the Control group (*n* = 30). The M-TAPA group received ultrasound-guided nerve block, while the Control group received a placebo injection. Postoperative pain was assessed using the numerical rating scale (NRS) over a 48-h period. Additional outcomes included opioid consumption and opioid-related side effects, such as nausea and vomiting.

**Results:**

The M-TAPA group had significantly lower NRS scores at all time points compared to the Control group, with the largest difference observed at 6 h postoperatively (4.27 ± 0.83 in the Control group vs. 2.19 ± 0.54 in the M-TAPA group). Repeated measures ANOVA revealed a significant interaction between time and treatment (*F* = 20.813, *p* < 0.001). Opioid consumption was reduced by 22% in the M-TAPA group over 48 h (*p* < 0.001), and the need for antiemetic drugs was significantly lower (*p* = 0.020). No M-TAPA-related complications were observed.

**Conclusion:**

M-TAPA was found to be an effective method for reducing postoperative pain and opioid consumption in patients undergoing LRCD.

**Clinical Trial Registration:**

www.chictr.org.cn.

## Introduction

Laparoscopic renal cyst decompression (LRCD) is a minimally invasive procedure commonly used to treat large renal cysts, but significant postoperative pain is often experienced (1, 2). Traditional analgesic methods, such as patient-controlled intravenous analgesia (PCIA) and epidural anesthesia, are associated with higher rates of complications, including urinary retention, nausea, vomiting, and respiratory depression (3, 4). These issues can prolong hospital stays, delay recovery, and compromise postoperative safety (5). As a result, postoperative pain management in LRCD has shifted toward regional nerve blocks, which offer more precise pain relief and fewer complications.

LRCD typically involves three incisions: one for the laparoscopic scope, located 2–3 cm above the iliac crest on the mid-axillary line, and two others, one near the costovertebral angle of the 12th rib and the other below the costal margin on the anterior axillary line (1). These incisions require regional blockade covering the anterior and lateral branches of the intercostal nerves from T8 to T12 (6). Thoracic paravertebral block (TPVB) has been shown to be an effective nerve block technique for postoperative pain management in nephrectomy patients (7). However, TPVB is technically challenging and may lead to serious complications such as pneumothorax and spinal anesthesia (8), while local infiltration anesthesia often fails to provide adequate analgesia.

To address these limitations, the modified thoracoabdominal nerve block through the perichondrial approach (M-TAPA) has been introduced as a new regional anesthesia method (9). M-TAPA, a type of fascial plane block, offers a safer and more effective option in clinical practice. It provides pain relief by blocking the anterior and lateral cutaneous branches of the intercostal nerves from T6 to T12, effectively covering the abdominal wall, including the incisions made during LRCD (9, 10). M-TAPA has primarily been used for surgeries involving anterior abdominal wall incisions, such as gastrectomy, gynecological procedures, and cholecystectomy (10–12). Although a recent case report demonstrated that M-TAPA provided adequate analgesia for a child undergoing right nephrectomy (13), its application in LRCD remains uncertain. The incisions in LRCD are located on the lateral abdominal wall, necessitating the blockade of the intercostal nerves from T8 to T12. While M-TAPA theoretically covers this area by blocking nerves from T6 to T12 (14, 15), variations in nerve block distribution and drug spread may result in inconsistent analgesia. Further investigation is warranted to assess the coverage, analgesic efficacy, and safety of M-TAPA for LRCD, given its potential advantages as a fascial plane block with improved safety and effectiveness in clinical settings.

This study aims to address these issues through a randomized, double-blind controlled trial designed to evaluate the effectiveness and safety of unilateral M-TAPA in postoperative analgesia following LRCD. By clarifying the role of M-TAPA in postoperative pain management and enhanced recovery, the findings of this study will provide new evidence for optimizing postoperative pain strategies in LRCD.

## Materials and methods

### Trial design

This randomized double-blind controlled trial was conducted in accordance with the Declaration of Helsinki and was approved by the Medical Ethics Committee of the First Affiliated Hospital of Chongqing Medical University (No. 2024–269). The trial was registered prospectively on the Chinese Clinical Trial Registry^1^ with the identifier ChiCTR2400085105 on May 31, 2024. Preoperatively, all patients received standardized education regarding the NRS pain assessment tool, where 0 indicated no pain, 1–3 indicated mild pain during movement or deep breathing that does not interfere with sleep, 4–6 indicated moderate pain present at rest that interferes with sleep, and 7–10 indicated severe pain preventing sleep with associated diaphoresis. Patients were also instructed on the proper use of patient-controlled analgesia (PCA) pumps (Jiangsu Renxian Medical Technology Co., Ltd., China). Written informed consent was obtained from all participants, and all clinical data were anonymized to ensure confidentiality throughout the study.

### Patients

Seventy patients undergoing unilateral LRCD at our hospital between Jun 2024 and October 2024 were recruited for the study. The first patient was enrolled on June 17, 2024. Inclusion criteria required participants to be 18–70 years of age, weigh 45–85 kg, have a body mass index (BMI) of 18–25 kg/m^2^, and be classified as American Society of Anesthesiologists (ASA) physical status I to III (16). Exclusion criteria included peripheral neuropathy, allergies to opioids or local anesthetics, history of substance or chronic opioid use, recent analgesic use (within 24 h), contraindications to nerve blocks (such as infection or coagulation disorders), or the need for conversion to open surgery.

### Grouping and randomization

Patients were transferred to the anesthesia preparation room approximately 1 h before surgery and underwent randomization. Group allocation to either the M-TAPA or Control group was determined using a computer-generated random number table, with assignments concealed in sealed envelopes to ensure blinding. All regional anesthesia procedures were managed by the Acute Pain Service (APS) team, with randomization information secured in sealed envelopes accessible only to team members performing the nerve blocks. Patients remained unaware of their group assignment, and identical dressings were applied to puncture sites in both groups to maintain blinding. Patients, surgeons, anesthesia care teams, and postoperative follow-up personnel remained blinded to group allocation throughout the entire perioperative period.

### Anesthesia intervention

After entering the operating room, all patients were continuously monitored for electrocardiography (ECG), peripheral oxygen saturation (SpO2), radial artery blood pressure, bispectral index (BIS), end-tidal carbon dioxide (PETCO2), and body temperature. General anesthesia was induced in both groups with intravenous midazolam (0.05 mg/kg), propofol (2 mg/kg), cisatracurium (0.2 mg/kg), and sufentanil (0.5 μg/kg). Anesthesia maintenance was achieved with propofol (4–6 mg/kg/h), remifentanil (0.02–0.2 μg/kg/min), and sevoflurane (0.5 minimum alveolar concentration). If blood pressure and heart rate increased by more than 20% from baseline after skin incision, additional sufentanil was administered and remifentanil infusion rate was adjusted. BIS values were maintained between 40 and 60 throughout the procedure. PETCO2 was kept between 35 and 45 mmHg, and core body temperature was maintained within the normal range during the entire surgical procedure.

### Surgical procedures

All surgeries were performed by three experienced surgeons using a standardized technique. The procedure involved laparoscopy-assisted renal cyst decompression, with pneumoperitoneum pressure maintained at 12 mmHg. Three endoscopic incisions were made: one above the iliac crest on the midaxillary line, one at the 12th rib angle, and another below the rib margin on the anterior axillary line. A drainage tube was placed at the incision above the iliac crest and was removed 24 h postoperatively. Conversion to open surgery was performed in cases of severe adhesion or significant intraoperative bleeding, as determined by the surgeon.

### Implementation of M-TAPA

**Figure 1:**
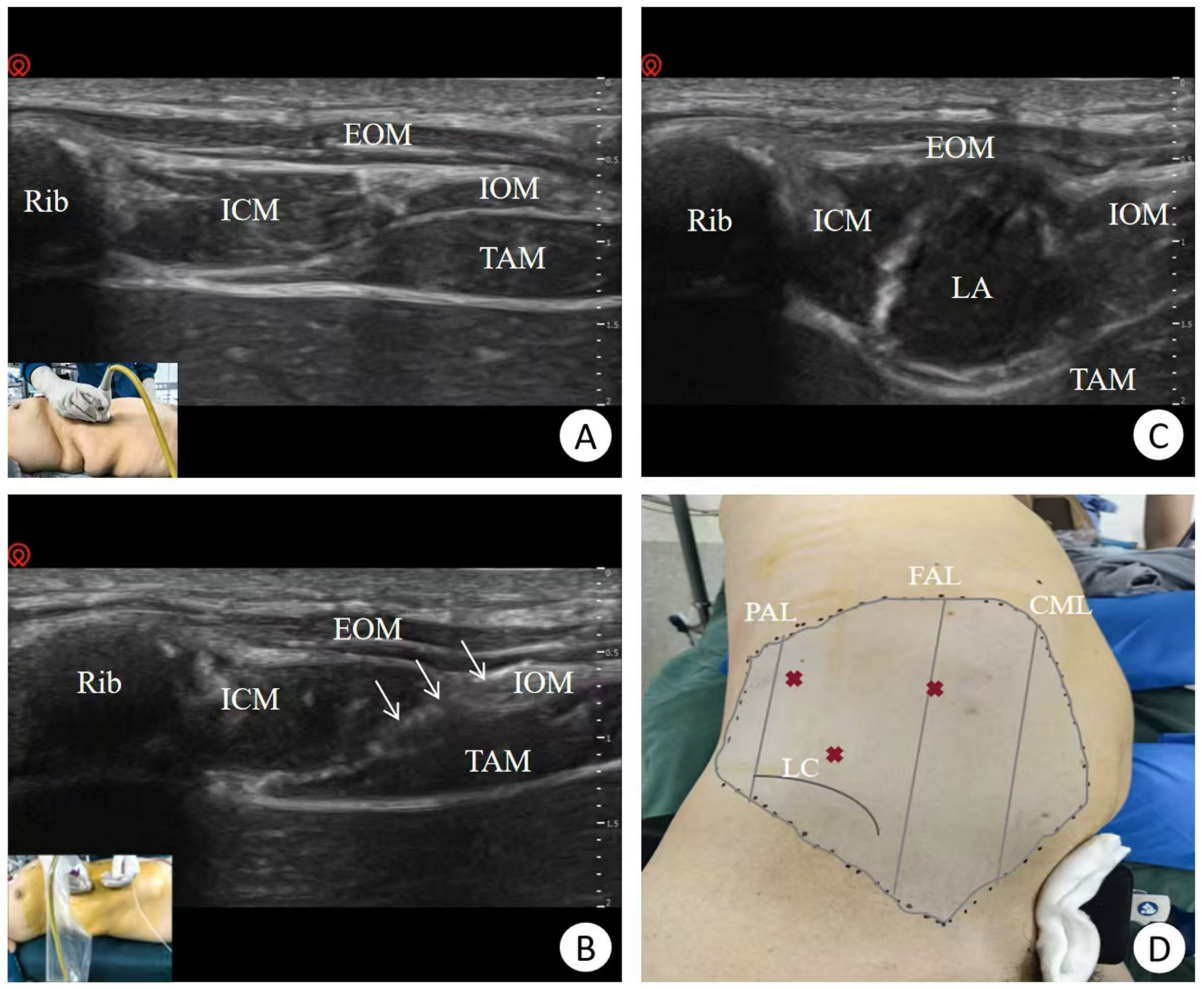
Ultrasound-guided M-TAPA via the perichondrial approach. **(A)** Ultrasound probe placement on the anterior axillary line showing the 10th rib and surrounding structures. **(B)** Ultrasound-guided nerve block puncture, the arrow indicates the visualization of needle. **(C)** Injection of local anesthetic solution. **(D)** Assessment of the nerve block range. PAL, Posterior axillary line; FAL, Front axillary line; CML, Clavicle midline; LC, Iliac crest; Rib, 10th rib; EOM, External oblique muscle; IOM, Internal oblique muscle; TAM, Transversus abdominis muscle; ICM, Intercostal muscle; LA, Local anesthetic.

TAPA was performed prior to anesthetic induction. The patient was positioned supine, and a 4.0–12.0 MHz linear ultrasound probe was placed along the anterior axillary line on the surgical side to identify the 10th rib. The 10th rib, along with the external oblique muscle (EOM), internal oblique muscle (IOM), transversus abdominis muscle (TAM), and intercostal muscle (ICM), were visualized (Figure 1A). Under ultrasound guidance (Navi; Shenzhen Wisonic Medical Technology Co, Ltd.), an experienced anesthesiologist used an in-plane technique to insert a 21G, 100 mm block needle (Echo Plus; Vygon) toward the cephalic end (Figure 1B). Once the needle tip was positioned on the surface of the TAM under the 10th rib, 30 mL of 0.25% ropivacaine was injected into the plane between the ICM and TAM in the M-TAPA group, or 30 mL of normal saline in the Control group (Figure 1C). Before injection, the fascial plane was confirmed with the “hydrodissection” technique to prevent complications such as organ injury or intravascular injection (17). Sensory block was assessed 30 min post-injection using a needle puncture test to evaluate the extent of the blockade (Figure 1D).

### Postoperative pain management

Postoperative analgesia was initiated following the completion of suturing, with all anesthetic agents discontinued. Patients received PCIA containing 100 μg sufentanil, 5 mg tropisetron, and 93 mL normal saline, with a total volume of 100 mL. The PCIA settings included a background infusion of 2 mL per hour, a bolus dose of 1 mL available upon patient demand, and a lockout interval of 15 min. A 1 mL bolus dose was administered if the NRS score exceeded 3, and if pain remained uncontrolled, 50–100 mg tramadol was given as rescue analgesia. To prevent postoperative nausea and vomiting (PONV), 2 mg tropisetron were administered intravenously if no contraindications were present.

### Outcome measurements

Sensory blockade in the lateral abdominal wall was assessed 30 min after M-TAPA by a researcher not involved in the surgery, using a needle puncture test. Sensory response was rated on a 3-point scale (0 = no pain, 1 = reduced pain, 2 = normal pain), with scores of 0 or 1 considered effective compared to normal shoulder sensation (11). Pain intensity was measured using the NRS at 1, 2, 4, 6, 12, 24, and 48 h postoperatively, reflecting the expected duration of ropivacaine’s effect. The primary outcome measure was postoperative pain intensity assessed using the numerical rating scale (NRS) between the M-TAPA and Control groups. Secondary outcome measures included analgesic consumption, rescue analgesia frequency, antiemetic use, time to first ambulation, time to first passage of flatus, and average length of hospital stay (ALOS). Opioid consumption during and after surgery, including sufentanil, remifentanil, and tramadol, was recorded and converted to morphine equivalents. PONV was evaluated using a descriptive scale (0 = none, 1 = mild nausea, 2 = moderate nausea, 3 = single vomiting, 4 = multiple vomiting). Rescue antiemetics were administered as needed for moderate to severe symptoms, and the rate of antiemetic use was recorded. Additionally, time to first ambulation, time to first passage of flatus, and average length of hospital stay (ALOS) were documented.

### Statistical analysis

Sample size was calculated using Power Analysis and Sample Size software, based on an assumed difference in postoperative NRS scores between groups (*δ*) of 1 and a standard deviation (*σ*) of 0.8 (12), with a significance level (*α*) of 0.05 and statistical power of 0.90. This calculation indicated a minimum of 15 participants per group.

The Kolmogorov–Smirnov test was applied to assess the normality of continuous data. Normally distributed data were expressed as mean ± standard deviation and compared using independent *t*-tests, while non-normally distributed data were presented as median (interquartile range) and analyzed using the Mann–Whitney U test. Categorical variables were reported as numbers (*n*) and percentages (%) and compared using the Chi-square test or Fisher’s exact test, as appropriate. For the analysis of NRS scores over time, repeated measures analysis of variance (ANOVA) was performed to evaluate the interaction between time and treatment. Statistical significance was set at *p* < 0.05. All analyses were performed using SPSS software (version 26, SPSS Inc., Chicago, IL, USA).

## Results

**Figure 2:**
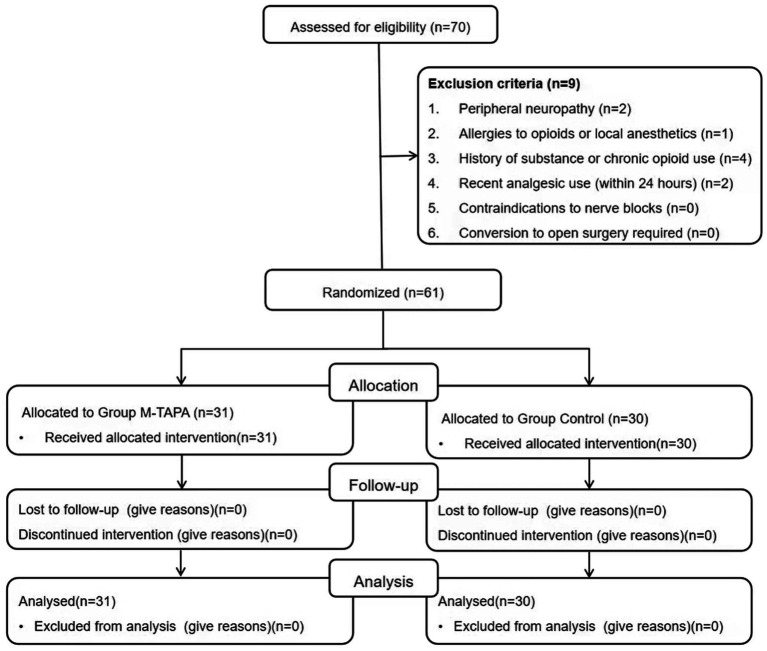
Flowchart illustrating the patient selection process for the study.

Out of the 70 patients initially enrolled, 61 were included in the final analysis, with 31 in the M-TAPA group and 30 in the Control group (Figure 2). Table 1 presents the baseline characteristics of patients in both groups.

**Table 1:** Baseline characteristics of M-TAPA and control groups.

Baseline characteristics	M-TAPA group (*n* = 31)	Control group (*n* = 30)
Age, years	59.13 ± 10.48	56.43 ± 9.38
Male, *n* (%)	18 (58.07)	16 (53.33)
BMI, kg/m^2^	24.10 ± 2.67	23.63 ± 2.99
Smoking history, *n* (%)	7 (22.58)	5 (16.67)
Surgical history, *n* (%)	4 (12.90)	3 (10.00)
Hemoglobin, g/L	131.42 ± 8.85	129.53 ± 9.67
ASA physical status ≥ III, *n* (%)	11 (35.48)	9 (30.00)

### Postoperative pain assessment

**Figure 3:**
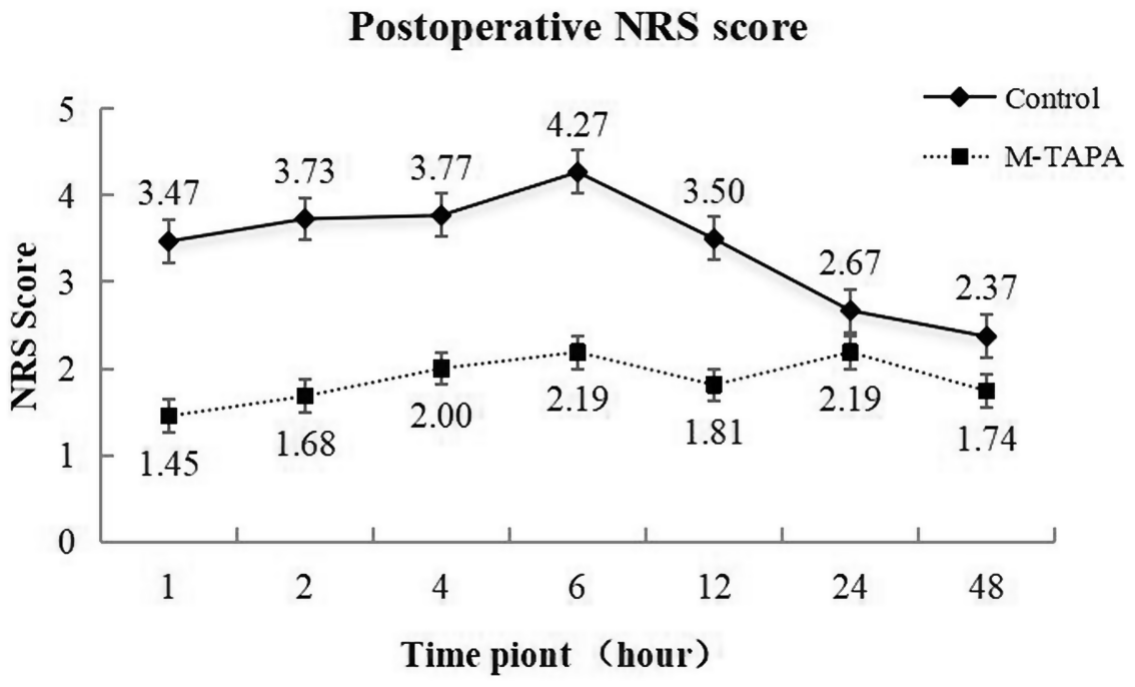
Trends in NRS scores at different time points over 48 h for the M-TAPA and Control groups.

The postoperative NRS scores for both the M-TAPA and Control groups over 48 h are presented in Figure 3. At all time points, the M-TAPA group demonstrated consistently lower NRS scores compared to the Control group. The highest score in the Control group was recorded at 6 h postoperatively (4.27 ± 0.83), while the M-TAPA group showed a significantly lower score (2.19 ± 0.54), representing the largest difference between the groups. Repeated measures ANOVA indicated significant changes in NRS scores over time within both groups (*F* = 24.483, *p* < 0.001). Between-group comparisons further revealed that the NRS scores in the M-TAPA group were significantly lower than those in the Control group (*F* = 197.657, *p* < 0.001). A significant interaction between time and treatment was observed (*F* = 20.813, *p* < 0.001), highlighting the strong analgesic effect of M-TAPA on reducing NRS scores compared to the Control group.

### Analgesic consumption

**Figure 4:**
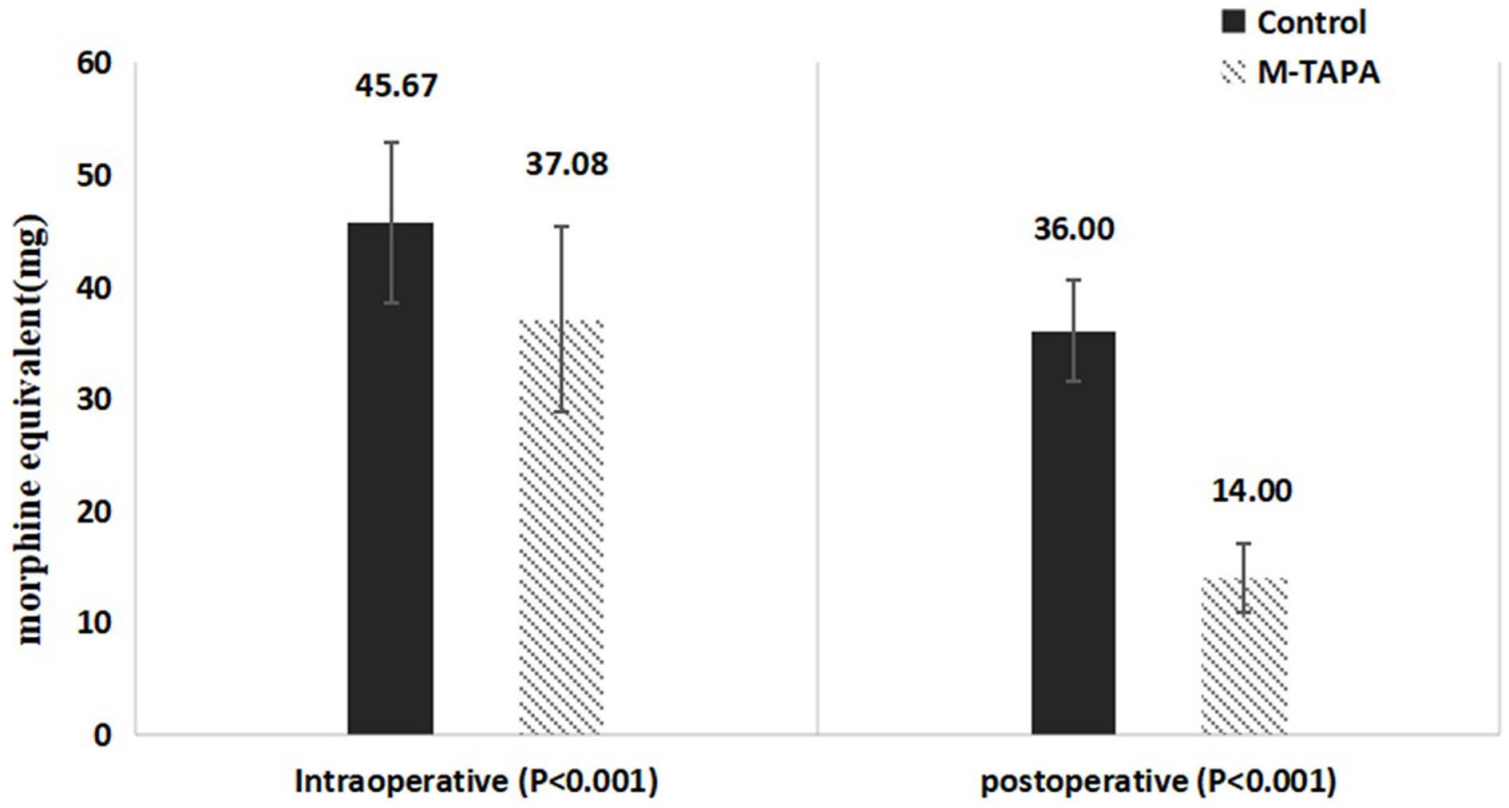
Comparison of intraoperative and postoperative analgesic consumption (morphine equivalents) between M-TAPA and control groups.

The Control group required significantly higher intraoperative and postoperative analgesic doses (in morphine equivalents of sufentanil, remifentanil, and tramadol) compared to the M-TAPA group. As shown in Figure 4, intraoperatively, the use of sufentanil and remifentanil (in morphine equivalents) was reduced by 8.59% in the M-TAPA group (*p* < 0.001). Over the 48-h postoperative period, the total analgesic consumption (in morphine equivalents of sufentanil and tramadol) was reduced by 22% in the M-TAPA group (*p* < 0.001).

### Perioperative outcomes

Table 2 outlines the key postoperative outcomes for the M-TAPA and Control groups. There were no significant differences between the groups in surgical duration, anesthesia time, or estimated blood loss (all *p*-values > 0.05). Patients in the M-TAPA group reported significantly lower NRS scores and required fewer rescue analgesic interventions compared to the Control group. The M-TAPA group also had a lower rate of antiemetic use. Recovery was faster in the M-TAPA group, as demonstrated by shorter times to first ambulation and first passage of flatus. Additionally, the ALOS was significantly reduced in the M-TAPA group compared to the control group (all *p*-values < 0.05).

**Table 2:** Perioperative outcomes between M-TAPA and control groups.

Perioperative outcomes	M-TAPA group (*n* = 31)	Control group (*n* = 30)	*p* value
Operation time, min	51.48 ± 8.90	47.37 ± 8.82	0.075^a^
Anesthesia time, min	70.68 ± 9.88	67.93 ± 7.96	0.238^a^
Blood loss, mL	55 (45, 100)	100 (80, 100)	0.122^c^
Average NRS score	1.87 ± 0.28	3.48 ± 0.49	<0.001^a^
Rescue analgesia frequency	0 (0, 0)	2 (0, 3)	<0.001^c^
Antiemetic use, *n* (%)	5 (16.13)	13 (43.33)	0.020^b^
First ambulation, h	22 (20, 24)	36 (26, 40)	<0.001^c^
First passage of flatus, h	18 (15.5, 22.0)	39 (30, 44)	<0.001^c^
ALOS, days	3 (3, 4)	4 (3, 5)	<0.001^c^

^a^for independent sample *t*-test, ^b^for chi-square test, and ^c^for Mann–Whitney U test.

## Discussion

The present study demonstrated that ultrasound-guided M-TAPA is an effective and safe method for providing postoperative analgesia to the unilateral anterior and lateral abdominal walls in patients undergoing LRCD. M-TAPA significantly reduced postoperative pain, as evidenced by lower NRS scores over the 48-h period. Furthermore, patients in the M-TAPA group showed a decreased need for opioid analgesics and antiemetic medications, indicating not only better pain control but also fewer opioid-related side effects. Early ambulation and gastrointestinal function recovery were also facilitated in the M-TAPA group, contributing to an overall faster postoperative recovery. Importantly, no complications related to the nerve block were observed, highlighting the safety profile of M-TAPA in this surgical setting.

Although LRCD is a minimally invasive procedure, significant postoperative pain can still arise from both the unilateral anterior and lateral abdominal walls, presenting a challenge for effective pain management (18). Multimodal analgesia, combined with peripheral nerve blocks, has become the preferred approach in such cases. This study evaluated the effectiveness of PCIA combined with M-TAPA for postoperative pain relief. M-TAPA, a novel ultrasound-guided regional anesthesia technique, provides comprehensive analgesia and extensive skin segment coverage following laparoscopic abdominal surgery (19). The local anesthetic administered beneath the 10th rib blocks the anterior and lateral cutaneous branches of the intercostal nerves, possibly by spreading within the space between the rib cartilage and the origin of the transversus abdominis muscle, known as the space between the intrathoracic fascia, diaphragm, and costophrenic recess (SEDIC) (20). Local anesthetic was injected into this space, bypassing the obstruction of the abdominal muscle line, to reach both the anterior branches of the thoracoabdominal nerve and the lateral cutaneous branches (20, 21), resulting in extensive blockade and effective relief of incision pain in the lateral abdominal wall. Previous reports have shown that a single injection of local anesthetic into the intrathoracic fascia below the rib cartilage can achieve multisegmental intercostal nerve block (22). In this study, the M-TAPA block provided sensory blockade from T6 to T12 in the lateral abdominal wall 30 min after injection, as confirmed by a needle puncture test, covering the three incisions used in LRCD surgery.

Acute pain following LRCD surgery typically occurs within the first 48 h. In this study, NRS scores remained low within 48 h after M-TAPA with 30 mL of 0.25% ropivacaine, suggesting that the prolonged analgesic effect may be related to the volume and concentration of the local anesthetic. Aikawa et al. (10) reported a case of laparoscopic sleeve gastrectomy managed with M-TAPA, where abdominal sensory blockade (T6-T12) was maintained for 48 h and disappeared by 56 h following the administration of 30 mL of 0.25% ropivacaine per side. Bilge et al. (12) found that M-TAPA effectively reduced postoperative pain scores and opioid consumption in patients undergoing laparoscopic cholecystectomy. Recent case reports have also demonstrated that M-TAPA provided adequate analgesia for a child undergoing right nephrectomy (13). In the present study, M-TAPA was administered before surgery, and a significant reduction in intraoperative sufentanil consumption was observed during LRCD. Postoperatively, there was a significant decrease in both NRS scores and the need for additional analgesics within 48 h, indicating that M-TAPA effectively provided pain relief for lateral abdominal wall surgeries.

In addition to pain, complications such as PONV caused by systemic intravenous analgesia, including PCIA, are significant factors that can hinder the rapid postoperative recovery of surgical patients. In this study, M-TAPA was associated with a lower number of PCIA presses and reduced use of antiemetic drugs, which contributed to shorter times to first ambulation, first passage of flatus, and a reduced ALOS. This can be attributed to the ability of nerve blockade to inhibit the transmission of pain impulses, prevent central sensitization, and reduce acute postoperative pain (23). Consequently, opioid use was decreased, promoting faster recovery of gastrointestinal function, reducing the duration of hospital stay, and facilitating a more rapid overall recovery after surgery (24, 25).

There are several limitations to this study. The sample size was calculated based on the expected impact of M-TAPA on postoperative pain scores to assess its effectiveness in multimodal analgesia. However, the current sample size was insufficient to evaluate less common adverse events, such as abdominal hematoma, vascular injury, or local anesthetic toxicity. Additionally, this study was conducted at a single center, and variations in local anesthetic diffusion among individuals could affect the outcomes. Therefore, further validation of these findings through large-scale, multicenter studies is needed to confirm the broader clinical application of M-TAPA.

## Conclusion

M-TAPA block effectively reduces postoperative pain, shortens hospital stay, and promotes early recovery in patients undergoing LRCD.

## Data Availability

The original contributions presented in the study are included in the article/Supplementary material, further inquiries can be directed to the corresponding author.

## Footnotes

^1^ www.chictr.org.cn
