# Supplementary material for: Ultrasound-guided modified thoracoabdominal nerve block for postoperative analgesia in laparoscopic renal cyst decompression: a randomized double-blind controlled trial
Source: Front Med (Lausanne). 2025 Jul 4;12:1582428. doi: 10.3389/fmed.2025.1582428 (PMC12271125; doi:10.3389/fmed.2025.1582428)
Supplement: Supplementary file 1 [file Data_Sheet_1.pdf]

				Table 1								Table 2								Figure 3				Figure 4	
No.in Control group	Age	Gender (Male=1, Female=2)	BMI	smoking history	Surgical history	Hemoglobin	ASA	Operation time	Anesthesia time	Blood loss	Average NRS score	Rescue analgesia frequency	Antiemetic use	First ambulation	First passage of flatus	ALOS	NRS at 1 h	NRS at 2 h	NRS at 4 h	NRS at 6 h	NRS at 12 h	NRS at 24 h	NRS at 48 h	Morphine equivalent (intraoperative)	Morphine equivalent (postoperative)
1	75	2	30.30	0	0	132	II	40	60	100	3.71	4	1	39	44	3	4	5	5	5	3	2	2	41.00	34.00
2	60	2	22.21	0	0	127	II	55	70	100	3.57	2	0	16	47	3	3	4	5	6	3	2	2	52.00	28.00
3	77	1	22.23	0	0	148	II	35	55	20	3.00	1	0	16	7	3	3	4	3	4	3	2	2	35.00	16.00
4	55	1	20.37	1	1	107	II	40	60	80	4.71	5	0	27	30	4	4	5	4	5	6	5	4	46.00	26.00
5	56	2	26.17	0	0	143	II	50	69	120	4.86	2	1	51	31	3	4	5	5	6	5	5	4	51.90	54.00
6	28	1	19.72	0	0	132	II	30	55	100	3.00	0	0	24	2	5	3	4	4	2	4	2	2	40.50	30.00
7	59	1	22.49	0	0	133	III	35	65	20	4.00	3	1	24	28	3	4	5	5	4	4	3	3	46.50	41.00
8	61	1	23.18	0	0	128	II	60	80	180	4.00	1	0	67	96	4	3	3	4	5	4	5	4	58.00	32.00
9	58	2	21.63	0	0	131	III	55	77	100	2.71	0	0	22	28	4	2	3	3	4	3	2	2	47.70	22.00
10	52	2	24.75	0	0	134	II	65	87	100	3.57	0	0	22	24	4	3	4	4	4	4	3	3	48.70	24.00
11	60	1	23.51	1	1	128	I	35	64	90	4.00	2	1	40	41	5	5	4	4	4	4	4	3	37.47	60.00
12	65	2	19.23	0	0	124	II	40	58	150	3.57	3	0	30	42	5	4	4	4	5	3	3	2	36.77	54.00
13	63	1	22.55	0	0	135	I	45	64	80	3.29	0	1	36	38	4	3	3	4	4	4	3	2	42.47	34.00
14	56	1	24.91	0	0	120	III	44	66	80	3.29	0	1	40	41	3	4	4	4	3	4	2	2	37.70	38.00
15	48	2	22.96	0	0	134	II	47	70	80	3.00	2	1	41	44	5	3	3	4	4	3	2	2	53.17	42.00
16	51	1	19.94	0	0	125	III	55	67	80	3.57	3	0	33	37	4	5	3	4	4	3	3	3	42.82	40.00
17	53	1	23.62	1	0	139	II	50	70	100	3.43	3	0	36	39	3	4	3	4	4	4	3	2	45.83	28.00
18	53	2	22.03	0	1	127	III	52	78	100	2.86	0	0	25	27	3	2	3	3	5	3	2	2	36.50	28.00
19	49	2	21.05	0	0	110	III	44	66	100	3.14	2	1	26	28	4	3	4	3	5	3	2	2	35.50	36.00
20	38	1	23.38	0	0	118	II	45	68	100	3.00	0	0	42	44	5	3	4	3	4	3	2	2	45.67	38.00
21	48	2	19.91	0	0	134	I	47	73	80	3.00	2	1	40	42	5	4	4	3	3	3	2	2	31.08	42.00
22	65	2	30.04	0	0	138	II	40	66	80	2.86	3	1	34	36	4	3	3	3	4	3	2	2	45.67	54.00
23	53	1	26.89	1	0	118	III	37	55	100	3.00	0	0	36	41	3	4	3	3	4	3	2	2	51.08	36.00
24	55	2	27.24	0	0	123	II	45	60	100	3.29	3	1	45	50	4	4	4	3	5	3	2	2	50.50	44.00
25	62	2	21.47	0	0	115	II	50	75	20	3.71	3	0	34	38	5	5	4	4	4	4	2	3	44.58	34.00
26	61	1	25.06	0	0	143	II	55	80	110	2.86	1	0	44	40	4	2	3	4	4	3	3	1	40.00	28.00
27	58	1	26.23	1	0	132	II	60	70	100	3.00	2	1	35	40	6	3	3	4	4	3	2	2	46.25	44.00
28	59	1	28.57	0	0	127	III	55	65	50	3.43	3	1	38	45	5	3	4	3	5	4	2	3	51.67	36.00
29	62	2	21.45	0	0	140	III	50	70	60	2.86	4	0	46	36	4	3	3	3	4	3	2	2	45.83	44.00
30	53	1	25.71	0	0	141	II	60	75	55	3.57	3	0	40	52	6	4	4	4	4	3	4	2	40.42	48.00

No.in M-TAPA group	Age	Gender (Male=1, Female=2)	BMI	smoking history	Surgical history	Hemoglobin	ASA	Operation time	Anesthesia time	Blood loss	Average NRS score	Rescue analgesia frequency	Antiemetic use	First ambulation	First passage of flatus	ALOS	NRS at 1 h	NRS at 2 h	NRS at 4 h	NRS at 6 h	NRS at 12 h	NRS at 24 h	NRS at 48 h	Morphine equivalent (intraoperative)	Morphine equivalent (postoperative)
1	72	1	26.99	1	0	130	I	37	55	50	1.57	0	0	22	18	2	1	1	2	2	1	2	2	35.5	12
2	55	2	24.44	0	0	123	II	44	67	50	1.71	0	1	23	20	3	1	1	2	2	2	2	2	31.7	16
3	68	1	29.41	0	0	110	II	45	68	10	1.57	0	1	25	17	3	1	2	2	2	2	1	1	36.8	18
4	67	1	24.09	0	0	134	I	50	74	180	2.57	0	0	24	23	6	1	2	3	2	3	4	3	37.4	16
5	52	2	22.46	0	1	119	III	45	57	100	1.29	0	0	24	16	2	0	1	1	2	2	2	1	30.7	10
6	57	2	21.22	0	0	123	II	47	65	100	1.57	0	0	48	15	4	2	1	2	3	1	1	1	36.5	12
7	54	1	31.14	0	0	131	II	55	78	100	2.00	0	0	24	27	4	2	3	3	2	2	1	1	41.5	12
8	46	1	26.23	1	0	138	III	59	79	100	1.86	0	0	20	6	3	1	2	2	2	2	2	2	36.58	16
9	54	2	23.24	0	0	136	II	65	80	50	1.86	0	1	23	22	2	1	2	2	1	3	2	2	36.67	18
10	69	1	22.32	1	0	133	II	63	85	100	1.57	0	0	10	20	2	1	1	1	2	2	2	2	37.08	10
11	67	1	22.58	0	0	141	III	55	68	10	2.00	0	0	22	38	2	1	2	2	3	2	2	2	35.67	16
12	61	1	27.82	0	1	129	III	56	77	10	1.86	0	1	22	17	3	1	2	2	1	2	3	2	41.42	16
13	53	2	25.22	0	0	117	I	57	70	100	2.14	0	0	24	22	3	2	2	2	2	2	3	2	38.17	8
14	60	1	18.75	0	0	129	III	44	74	80	1.71	0	0	20	24	4	1	1	2	2	2	2	2	48.63	16
15	68	1	24.11	1	0	135	I	45	66	50	2.00	0	0	21	1	4	2	2	2	3	2	2	1	32.7	14
16	61	1	23.45	0	0	133	II	47	59	50	2.00	0	0	18	10	3	2	2	2	2	2	2	2	41.88	8
17	58	1	24.80	1	1	144	III	36	50	200	2.00	0	0	16	12	4	1	2	2	2	2	3	2	35.83	16
18	80	2	21.51	0	0	124	III	37	52	200	1.71	0	0	15	8	3	1	2	2	3	1	2	1	40.2	16
19	58	2	22.77	0	0	125	II	44	68	120	2.14	0	0	16	16	3	2	2	3	2	1	3	2	41.8	10
20	55	2	23.60	0	1	134	I	45	70	100	2.00	0	0	20	18	2	2	2	2	2	2	2	2	37	11
21	58	1	23.45	0	0	118	II	47	80	20	2.43	0	0	24	17	4	2	3	2	3	2	3	2	48	12
22	40	1	25.06	1	0	144	I	55	77	200	1.86	0	0	10	15	4	1	1	2	2	2	3	2	47.7	13
23	55	2	23.53	0	0	142	II	57	83	100	2.00	0	1	24	29	3	2	2	2	2	2	2	2	33.3	14
24	77	2	28.57	0	0	129	III	46	59	50	1.71	0	0	24	20	3	1	1	2	2	2	2	2	35.9	16
25	58	1	22.60	0	0	136	II	50	65	50	2.00	0	0	24	20	3	2	1	2	3	2	2	2	36.5	12
26	68	1	26.57	1	0	134	II	65	75	20	2.00	0	0	18	24	3	2	2	2	3	2	2	1	47.5	14
27	28	2	21.22	0	0	145	II	65	85	20	1.71	0	0	20	25	3	1	2	1	2	1	3	2	43.5	12
28	65	2	22.06	0	0	122	III	60	80	20	1.14	0	0	22	18	2	1	1	1	2	1	1	1	43	16
29	50	1	21.30	0	0	138	III	70	85	55	2.00	0	0	24	20	3	2	2	2	3	1	2	2	48.5	12
30	66	1	24.09	0	0	142	III	50	65	40	2.00	0	0	20	15	4	3	1	2	2	1	3	2	36.5	14
31	53	2	22.58	0	0	136	II	55	75	60	1.86	0	0	23	20	2	2	1	3	2	2	2	1	37.5	15
